# In regard to the article ‘Effectiveness of robust optimization in volumetric modulation arc therapy using 6 and 10 MV flattening filter-free beam therapy planning for lung stereotactic body radiation therapy with a breath-hold technique,’ Vol. 61, No. 4, 2020

**DOI:** 10.1093/jrr/rrab019

**Published:** 2021-06-22

**Authors:** Priyanka Agarwal, Rajesh Kinhikar

**Affiliations:** 1 Medical Physicist, Homi Bhabha Cancer hospital, Varanasi, UP 221005, India; 2 Medical Physicist, Tata Tata Memorial Hospital, Mumbai, Maharashtra 400012, India

The article entitled ‘Effectiveness of robust optimization in volumetric modulation arc therapy using 6 and 10 MV flattening filter-free beam therapy planning for lung stereotactic body radiation therapy with a breath-hold technique’ published in the *Journal of Radiation Research*, Vol. 61, No. 4, 2020 is well written and informative. Thanks to the authors.

In their article, Miura *et al.* generated four volumetric modulated arc therapy (VMAT) plans for all 10 lung stereotactic body radiation therapy (SBRT) patients using breath-holding techniques with 6X-FFF and 10X-FFF beams, and total 40 plans were evaluated for the study, namely, an optimized plan based on planning target volume (PTV) margin, and the other one robust optimization based on internal target volume (ITV) with setup uncertainties. The total dose 42 Gy was prescribed in four fractions for each plan and the study was concluded in favor of 10X-FFF for robust optimization based on ITV with setup uncertainties.

Vassiliev *et al.* [1] reported that lung tumor under-dosing is mainly affected by two parameters, the smaller volume of tumor and lower lung density, with an increase in photon energy. The authors elaborated that the tumor periphery under-dosage is caused by electronic disequilibrium. The cause of this disequilibrium is the sharp density gradient between tissue density and lung tumors. In this case, the rate of electron production inside and outside the tumor will not be the same and results in disequilibrium and tumor surface under-dosing. The second reason is the large range of electrons in lower lung density. Consequently, the electron is not produced adjacent to the tumor surface but farther upstream. This electronic disequilibrium is more pronounced for a small volume tumor. In some cases, it may under-dose the tumor by 10% of the prescription dose, or more. Therefore, as the photon energy increases, in the case of small lung tumor volume located in the air cavity, the tumor under-dose cannot be negligible.

In their article, Miura *et al.* have found the plan with robust optimization based on ITV was better compared to an optimized plan based on PTV for their corresponding energies. The authors have shown a statistically significant difference (*p* < 0.05) among all four kinds of the plan for ITV, PTV and organs at risk (OAR). The ITV D99%, D98% were observed to be statistically significantly different for robust 6X-FFF and 10X-FFF and found less dose for 10X-FFF. The mean dose received by lung (in cGy), V20% and V5% (in percent) were 306.6 cGy, 3.8% and 14.2% respectively for robust 6X-FFF. In contrast, the same for robust 10X-FFF were 336.1 cGy, 4.4% and 16.3% respectively, and were observed to be statistically significantly different. When the PTV was situated in an offset position, the dose pattern obtained was similar.

There are many articles in various journals mentioned in Table 1, in which the authors have compared 6XFFF and 10XFFF beam dosimetrically for lung SBRT. In all the articles, the OARs were better spared by using 6XFFF with the same PTV coverage compared to 10XFFF. As per Miura *et al.*, the main cause to select the robust 10X-FFF beam for planning was due to the short delivery time, resulting in a reduction of intra-fractional motion during treatment. However, despite the 10XFFF beam offering a short delivery time, beam-on time also depends on dose per fraction. Lu *et al.* [2] have compared 6XFFF and 10XFFF beam with different dose fractionation schemes that were 4X12, 3X18 and 1X34 Gy. The plans were compared mainly in terms of treatment efficiency, and OAR-sparing. In the article, the authors found approximately a 15% dose reduction in each OAR with each fractionation scheme using 6XFFF compared to 10XFFF. Also, the reduced beam-on time by using 10XFFF was 0.7, 1.3 and 2.8 min, compared to 6XFFF with the fractionation scheme 4X12, 3X18 and 1X34 Gy, respectively. Lu *et al.* [2] found the same tumor control probability (TCP) for both energies. But, normal tissue complication probability (NTCP) for 6XFFF was significantly reduced for lung and chest wall. The percentage of NTCP reduction for lungs were 10.3 ± 4.5, 9.9 ± 3.6% and 7.4 ± 2.2% for 4X12, 3X18 and 1X34 Gy, respectively. The same for the chest wall was found to be 11.9 ± 13.7%, 10.0 ± 8.8% and 2.6 ± 2.5%, respectively. In this article, we recommend 6XFFF for 4X12, 3X18 and 10XFFF for 1X34 Gy, while balancing the treatment efficiency and OAR-sparing.

**Table 1:** Dosimetry comparisons between 6XFFF and 10XFFF beam in various publications

**Author names**	**Beam energy**	**OARs doses**	**No of patients**	**Authors’ conclusions**
Durmus *et al.* (2018) [3]	6XFFF vs 10XFFF	3.5% lower dose for Ipsi-lung, high dose spillage for 6XFFF compared to 10XFFF	16	6XFFF
Hrbacek *et al.* (2014) [4]	6XFF, 6XFFF & 10XFFF	Lung V20 & V12.5Gy, 5.5% and 4.5% lower for 6XFFF, compared to 10XFFF	11	6XFFF
Lu *et al.* (2015) [2]	6XFFF vs 10XFFF	10% lower dose for lung and chest wall for 6XFFF compared to 10XFFF	12	6XFFF

**Table 2:** The appeared radiation toxicity of Lung SBRT patients in various studies

**Author names**	**Radiation toxicity**	**Follow up time**	**Dose prescription**	**No of patients**
Arnett *et al.* (2019) [5]	Grade ≥ 3 pneumonitis (0.97%), Grade 3 Myocardial Infarction (1.9%), Grade 3 pneumonitis (3.9%)	50 months	50Gy/5# or 48Gy/4#	103
Haasbeak *et al.* (2011) [6]	Late toxicity, Grade ≥ 1 (17%), Grade ≥ 2 (14%) and Grade ≥ 3 (6%), in which dyspnea, chest wall pain, fatigue and one patient with radiation dermatitis.	35 months	60Gy/8#	63
Lin. *et al.* (2019) [7]	Grade 1–2 (38.6%) and Grade 3 (1.4%) pneumonitis, dyspnea (10%), chest pain (5.7%), fatigue (5.7%)	24.9 months	50Gy/5# or 60Gy/8#	70
Zhao *et al.* (2020) [8]	Dyspnea (3.1%), pneumonitis (1%), Hemoptysis (1%)	22.9 months	60Gy/8#	98
Nicosia *et al.* (2019) [9]	Grade 1 (4.6%), Grade 2 (15.9%), Grade 3 (4.6%) pneumonitis, Grade 2 (2.3%) esophagitis and late toxicity Grade 1 (27.3%), Grade 2 (11.4%), Grade 3 (2.3%) lung fibrosis	34 months	30Gy/1#	44
Park *et al.* (2015) [10], for Peripheral lung tumor	Acute Grade 2 pneumonitis, fatigue, chest wall pain, esophagitis, dermatitis and Late Grade 2 toxicity same	21.4 months	54Gy/3#	140
Park *et al.* (2015) [10], for Central lung tumor	Acute Grade 2 pneumonitis, fatigue, chest wall pain, esophagitis and Late Grade 2 toxicity same	21.4 months	50Gy/4# or 50Gy/5#	111

Table 2 represents the radiation toxicity of lung SBRT patients in various studies. From the table, it is obvious that the main radiation toxicity appears as pneumonitis, myocardial infarction and in ribs instead of skin. If the tumor location is peripheral, it is possible that skin toxicity may appear. Park *et al.* [10] compared the radiation toxicity between centrally located lung tumors (for 111 patients) and peripheral lung tumors (for 140 patients) and found that in a medium follow-up, only three patients suffered dermatitis radiation toxicity in cases of peripheral tumor location.

For the clinical acceptance of planning and better clinical outcome, the As Low As Reasonably Achievable (ALARA) principle should be followed for OARs, such as lungs, heart and the ches wall.

We are in the opinion that for lung SBRT, robust 6X-FFF may be an appropriate and optimal choice of the beam from all the aspects (tumor coverage, OARs doses, integral dose).

## CONFLICT OF INTEREST

The authors declare they have no conflict of interest.
